# Role of the Crystallographic Phase of NiTi Rotary Instruments in Determining Their Torsional Resistance during Different Bending Conditions

**DOI:** 10.3390/ma14216324

**Published:** 2021-10-23

**Authors:** Alessio Zanza, Marco Seracchiani, Rodolfo Reda, Dario Di Nardo, Gianluca Gambarini, Luca Testarelli

**Affiliations:** Department of Oral and Maxillo-Facial Sciences, Sapienza University of Rome, 00161 Rome, Italy; ale.zanza@gmail.com (A.Z.); marco.seracchiani@uniroma1.it (M.S.); dario.dinardo@uniroma1.it (D.D.N.); gianluca.gambarini@uniroma1.it (G.G.); luca.testarelli@uniroma1.it (L.T.)

**Keywords:** crystallographic phase, endodontics, nickel-titanium, root canal treatment, torsional stress

## Abstract

The aim of this study was to assess the role of the crystallographic phase of Nickel-titanium (NiTi) rotary instruments in determining their torsional resistance during different bending conditions, such as different degrees and angles of curvature. 200 F-One 20.04 instruments (Fanta Dental, Shanghai, China) were used, 100 austenitic instruments and 100 martensitic instruments. Each group was divided in 5 subgroups according to the different bending conditions (straight canal, 90° or 60° of curvature degrees and 3 mm or 5 mm of radius of curvature). The static torsional test was performed by using a device composed of an electric motor capable of recording torque values (N·cm); a vice used to secure the instruments at 3 mm from the tip; and artificial canals, which allow instruments to remain flexed during test. Each instrument was rotated at 500 rpm with a torque limit set to 5.5 Ncm until its fracture. Torque at Fracture (TtF) was registered. A scanning electron microscopy (SEM) observation was conducted. The collected data confirm that an increase in the angle of curvature and a decrease in the radius of curvature of the artificial canals lead to an increase of TtF values with a statistically significant difference (*p* < 0.05), both in the austenitic and martensitic groups. Regarding the comparison between austenitic and martensitic groups in the same bending condition, a statistically significant difference was found only when the torsional test was performed in the canals with the degrees of curvature of 90° and the radius of curvature of 3 mm and 5 mm, with the austenitic instruments showing a higher TtF than the martensitic ones. In conclusion, it can be stated that the crystallographic phase influences the maximum torque at fracture when the instruments are subjected to severe bending and that the radius of curvature significantly influences their torsional resistance.

## 1. Introduction

As demonstrated by several research studies, root canal anatomy significantly influences the mechanical behavior of nickel-titanium (NiTi) rotary instruments, facilitating structural stress generation [1,2,3]. The most remarkable characteristics of root canal geometry are undoubtedly the diameter and the curvature configuration [4].

Regarding curvature configuration, Schneider (1971) proposed the degree of root canal curvature as the main parameter for assessing curvature severity [5]. In 1997, Pruett et al. proposed a new method of canal curvature evaluation that addressed both the angle and the abruptness of curvature, which considered the degrees and the radius of curvature, demonstrating that the location of the curvature along the canal could influence the resulting curvature angle. According to this, it has been proven that the cyclic fatigue resistance of NiTi rotary instruments significantly increases as the radius of curvature increases and when increasing the angle of curvature, the cyclic fatigue resistance significantly decreases [4]. Nevertheless, there are no studies that simultaneously evaluate the mutual influence of different angle and radius of curvature on the torsional resistance of NiTi rotary instruments.

During the shaping procedures of root canals with small diameters, NiTi rotary instruments are subjected to higher torsional stress compared with the instrumentation of larger canals, increasing the likelihood of intracanal torsional failure [6]. Fracture due to torsion can occur when a part of the instrument binds in the canal while the handpiece continues to rotate at a constant speed. When the elastic/plastic limit of the instrument is exceeded, fracture occurs [7]. The parameters involved in determining the torsional resistance of NiTi rotary instruments have been thoroughly investigated. Recent studies have stated the importance of the cross-sectional design in determining the torsional resistance of NiTi rotary instruments [8,9], highlighting the importance of the polar moment of inertia over metal mass, volume, diameter, and inner core radius [10]. However, the transversal characteristics of NiTi rotary instruments are not the only factors that affect the torsional resistance, since it has been showed that the longitudinal features such as pitch, number of threads, helix angle, and the length of the instrument can also affect it [9,11].

In order to decrease likelihood of intracanal separation due to cyclic fatigue, manufacturers have developed particular heat-treatments for nickel-titanium alloys, allowing endodontic instruments to be in the martensitic phase at the ambient temperature. In light of this, numerous studies have been conducted in order to investigate the metallurgic behavior of austenitic and martensitic instruments [12,13]. The nickel-titanium alloy is an equiatomic alloy existing in two different phases, which differ from each other in terms of their crystallographic structure: the austenite and the martensite. The first phase is characterized by stiffness, hardness, and increased superelasticity. On the contrary, the martensitic phase is characterized by ductility, softness, deformability, and a shape memory effect, that allow endodontic instruments to better withstand flexural stress and cyclic fatigue [12,14].

The crystallographic phase of the NiTi alloy, the transition temperature range, and the effect of heat-treatments can be conveniently studied using the differential scanning calorimetry (DSC), in which the difference in thermal power supplied to a test specimen and an inert control specimen heated at the same rate is measured [15].

In a recent study, Seracchiani et al. investigated the relationship between the flexural stress and the torsional resistance of martensitic NiTi instruments, stating that the torque at fractures values increase when instruments are subjected to flexural stress [16]. Nevertheless, no data are available regarding the torsional behavior of austenitic instruments in the presence of flexural stress. Moreover, in the aforementioned study, the authors only considered the curvature degrees with a constant radius of curvature, and did not investigate its influence on stress distribution.

On the basis of the above, the purpose of this study was two-fold:

1. Investigate the relationship between the flexural stress and the torsional resistance in austenitic rotary instruments and elucidate if there are any differences with martensitic ones.
2. Investigate the influence of different degrees and radius of curvature on torsional resistance of martensitic and austenitic rotary instruments when subjected to flexural stress.

According to this, the null hypothesis was that the torsional resistance of endodontic instruments with the same morphological characteristics and different crystallographic organization does not vary in case of different bending conditions (different degrees and angles of curvature).

## 2. Materials and Methods

To conduct this study on the torsional resistance of two different NiTi instruments, the sample size was calculated using G*Power software (Version 3.1.9.6., Heinrich-Heine-Universität Düsseldorf, Düsseldorf, Germany), assuming a standard deviation of 0.04 Ncm and a torque value of 0.22 N·cm as the expected difference. It was calculated that 20 instruments were required for each group to have a reasonable certainty that the statistical power will be 0.8 (1-β value) and the significance level will be 0.05 (α value).

Thus, 200 instruments F-One 20.04 (Fanta Dental, Shanghai, China) were used in this study: 100 austenitic instruments (group A) and 100 martensitic instruments (group B). The instruments of both groups share the same dimensional characteristics and differ only for their crystallographic phase. The group A instruments are characterized by a conventional NiTi alloy, and they were produced specifically for this research by the manufacturer (Fanta Dental, Shanghai, China), while those belonging to the group B are characterized by the heat-treatment AF™-R Wire and they are available on the market.

Before testing, each instrument was inspected through stereoscopic magnification ×20 (Carl Zeiss Micro-imaging, Göttingen, Germany) in order to evidence manufacturing defects and none of them was discarded.

### 2.1. Static Torsional Test

The torsional test device used in this research was validated in previously published studies [16,17]. It is composed of an electric motor equipped with a 1:1 handpiece (Kavo, Biberach, Germany) capable of recording torque values (N·cm) every 1/10 of a second; a vice used to firmly secure the instruments at 3 mm from the tip; and artificial stainless steel (SS) canals, which allow instruments to remain flexed during test procedures.

**Figure 1:**
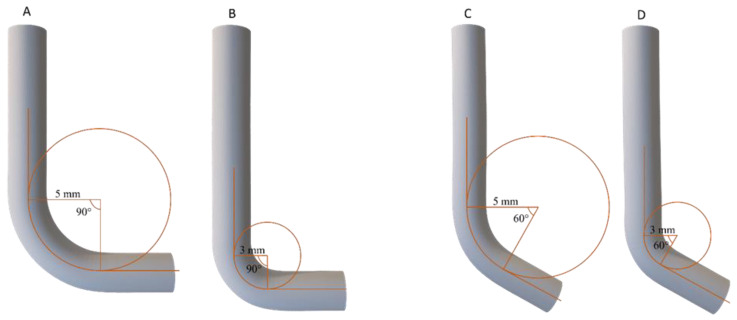
Schematic representation of the four curved artificial canals with different angles and radii of curvature used in the torsional tests, which allowed instruments to remain flexed during test procedures. (**A**) SS canal with the radius of curvature of 5 mm and the degrees of curvature of 90°; (**B**) SS canal with the radius of curvature of 3 mm and the degrees of curvature of 90°; (**C**) SS canal with the radius of curvature of 5 mm and degrees of curvature of 60°; (**D**) SS canal with the radius of curvature of 3 mm and the degrees of curvature of 60°.

In order to differentiate flexural stress conditions during tests, four different SS curved canals and 1 straight canal were used (Figure 1). They differ in terms of radius and angle of curvature as follows: 90° curvature with 5 mm radius, 90° curvature with 3 mm radius, 60° curvature with 5 mm radius, 60° curvature with 3 mm radius, and a straight canal. Each artificial SS canal was tested by freely rotating instruments inside it, with the aim to ensure the absence of any intrinsic ability of the SS canals to generate friction stress during instrument rotation. During this pre-test, the motor did not record any values (sensitivity of 0.01 N·cm); thus, the torque developed was considered null for each artificial canal.

Each instrument was rotated inside the selected artificial SS canal at 500 rpm with a torque limit set to 5.5 Ncm until the tip fracture. Torque at Fracture (TtF) was recorded, and fractured fragments were collected and measured with a digital caliper (0.01 mm of sensitivity) in order to evaluate the quality of the performed tests.

### 2.2. Groups and Subgroups Determination

According to the different flexural testing conditions, both groups A and B were divided in 5 subgroups (n = 20) as follows:

1. Group A1: 20 austenitic instruments rotated in a 60° curvature with 5 mm radius;
2. Group A2: 20 austenitic instruments rotated in a 60° curvature with 3 mm radius;
3. Group A3: 20 austenitic instruments rotated in a 90° curvature with 5 mm radius;
4. Group A4: 20 austenitic instruments rotated in a 90° curvature with 3 mm radius;
5. Group A5: 20 austenitic instruments rotated in a straight canal;
6. Group B1: 20 martensitic instruments rotated in a 60° curvature with 5 mm radius;
7. Group B2: 20 martensitic instruments rotated in a 60° curvature with 3 mm radius;
8. Group B3: 20 martensitic instruments rotated in a 90° curvature with 5 mm radius;
9. Group B4: 20 martensitic instruments rotated in a 90° curvature with 3 mm radius
10. Group B5: 20 martensitic instruments rotated in a straight canal.

### 2.3. Statistical Analysis

The standard deviation and mean values for TtF and fragment length (FL) were calculated and statistical analyzed using one-way analysis of variance with the Bonferroni correction for multiple comparisons across the subgroups. Parametric tests were adopted after performing a visual inspection of the distribution histograms, which confirmed the normal distribution of the analyzed data. The significance was set at the 95% confidence level.

### 2.4. Scanning Electron Microscopy

A scanning electron microscopy (SEM) observation was conducted with the aim to evaluate the topographic features of the fractured surface of the tested NiTi rotary instruments and to confirm the fracture due to torsion. Five fractured NiTi rotary instruments for each group (A and B) were randomly selected and then observed under a scanning electron microscope (FEI QUANTA 400, FEI Company, Hillsboro, OR, USA) and a fractographic analysis was performed.

## 3. Results

All results regarding TtF and FL according to the different bending conditions and the different crystallographic phases are summarized in Table 1.

### 3.1. Torque at Fracture (TtF)

According to the data and the statistical analysis, all subgroups (both for group A and group B), characterized by the torsional test performed in curved SS canals, showed higher TtF values compared to the subgroup A5 and the subgroup B5 in the straight canal (respectively of 0.42 ± 0.07 Ncm and 0.45 ± 0.02 Ncm), with a statistically significant difference between all subgroups and their respective subgroups A5 and B5 (*p* < 0.05).

Regarding the austenitic group (Group A), it has been pointed out that increasing the angle of curvature and reducing the radius of curvature of the SS canal during the torsional test, the TtF values increase with a statistically significant difference (*p* < 0.05). In other words, a statistically significant difference (*p* < 0.05) between the subgroups A1 and A2, A2 and A3, and A3 and A4 (Table 1) was pointed out.

The same mechanical behavior was detected for the martensitic group (Group B), with a statistically significant difference (*p* < 0.05) between the subgroups B1 and B2, B2 and B3, and B3 and B4 (Table 1).

Regarding the comparison between the austenitic and the martensitic subgroups in the same bending conditions, no statically significant difference (*p* > 0.05) was detected for the subgroups A5 and B5, A1 and B1, and A2 and B2, while a statistically significant difference (*p* < 0.05) was found for the subgroups A3 and B3, and A4 and B4 (Table 1).

### 3.2. Fragment Length (FL) Measurement

Regarding FL, no statistically significant differences (*p* > 0.05) between all subgroups were found, with mean values and standard deviations summarized in Table 1. These results evidenced the quality and the reproducibility of the test.

### 3.3. Scanning Electron Microscopy Observation and Fractographic Analysis

**Figure 2:**
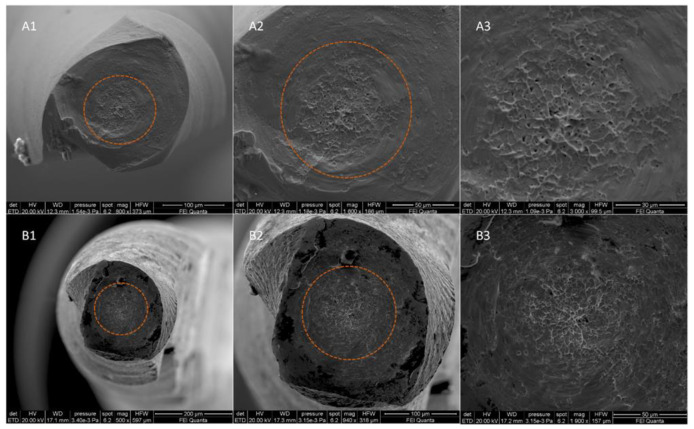
SEM images of fractured surface of two samples of F-One 20.04 in a transversal view at different magnifications after torsional fatigue testing. Images (**A1**–**A3**) represent SEM captures of an austenitic instrument, while images (**B1**–**B3**) represent SEM captures of a martensitic instrument. All acquisition specifications are shown in the black box below each image. The concentric circular abrasion marks and fibrous dimples near the center of rotation are evidenced by the orange circumferential lines.

The SEM observation and the fractographic analysis of the fractured instruments showed the typical features of fracture due to torsional load, with concentric circular abrasion marks and fibrous dimples near the center of rotation (Figure 2).

## 4. Discussion

During root canal shaping, NiTi endodontic rotary instruments are subjected to both flexural and torsional stresses due to their rotation and cutting action inside a curved canal. Despite this, only recently has attention been focused on the combined interaction of these two phenomena [16]. Previously, there was a tendency to consider flexural and torsional stresses as two distinct factors, not considering their reciprocal influence [18,19,20,21,22]. Nevertheless, first Seracchiani et al. recently demonstrated the relationship between the torsional resistance of NiTi rotary instruments and the bending moment acting on them, stating that when increasing the flexural loading, the torsional resistance of the bent instruments increases as well. Thus, the torque at fracture registered during static torsional test performed in curved canals is higher than in straight canals [16].

The physical rational behind it is that the total torque registered by endodontic motors is the driving torque resulting from the dentinal cutting and the bending moment acting on the flexed instrument, and it can be described with the following formula:

$$M=\sqrt{M_{b}^{2}+\frac{3}{4}T^{2}}$$

where *M* is the total torque, *M_b_* is the bending moment acting on the instrument and *T* is the twisting moment. Moreover, *M_b_* is defined as $M_{b}=k\cdot E_{y}\cdot I$, where *k* is 1/radius of curvature, *I* is the polar moment of inertia, and *E_y_* is the Young’s modulus that is an intrinsic property of the alloy that differs in case of different crystallographic phase (martensite and austenite) [16]. According to this, *M_b_* is influenced by both the radius and the angle of curvature, and the crystallographic phase of the alloy.

Nevertheless, as mentioned above, there are no studies that investigate the role of crystallographic phase and the different radius of curvature in modifying the torsional resistance of NiTi rotary instruments during different bending conditions.

Accordingly, the aim of this research was to assess in vitro the influence of these factors on the torsional resistance, comparing two instruments sharing the same characteristics except for their crystallographic phase, tested in different bending conditions.

As mentioned above, the torsional resistance was evaluated using a custom-made device and a methodology already validated in a previously published article [16]. However, two main differences could be observed in comparison to the ISO 3630-1 test. The first one is the rotational speed, which according to the ISO test should be set to 2 rpm; however, it has been stated that it did does influence the torsional resistance [23]. The second one is the introduced bending stress that it is not provided for the ISO test, but is fundamental for the aim of the study.

The F-One Blue 20.04 (Fanta, Shanghai, China) instruments were selected for this research. These instruments are characterized by a flat design, which reducing the metal mass along the instrument leads to an increase of the cyclic fatigue resistance [24,25].

According to the results, it can be stated that for both austenitic and martensitic instruments, when increasing the angle of curvature and reducing the radius of curvature of the artificial canal during the torsional test, the TtF values increase as well. In other words, when increasing the flexural stress to which an instrument is subjected, its torsional resistance increases as well—the higher the flexural resistance of an instrument, the more its torsional resistance increases. These finding are consistent with those of previous research and clearly follow the mechanical behavior described in the aforementioned formula [16,17]. In fact, reducing the radius of curvature and increasing the angle of curvature leads the value of the bending moment (*M_b_*) to increase, thereby increasing the total torque value (*M*).

Regarding the comparison between the instruments with different crystallographic phase tested in the same bending conditions, no statistically significant difference (*p* > 0.05) was detected for the subgroups tested in the straight canal and in the canals with 60° of curvature and radius of curvature of 3 mm and 5 mm. On the contrary, the results pointed out a statistically significant difference (*p* < 0.05) between the subgroups tested in the canals with 90° of curvature and radius of curvature of 3 mm and 5 mm. These results were not totally expected considering that austenitic instruments have a higher Young’s modulus than martensitic ones, and, thus, the value of the bending moment should be higher in the case of comparable instruments characteristics, such as tip diameter, taper, and cross sectional design [26]. For these reasons, a higher TtF should be expected even in the torsional tests performed in the canals of 60° of curvature, since in the case of same curvature characteristics, the austenitic instruments should be subjected to higher bending moment than martensitic ones, since they worsen withstand flexural stresses; thus, the resulting torque (*M*) should be higher.

Accordingly, it can be stated that a significative difference in terms of torsional resistance between a martensitic and an austenitic instrument can be found just in the case of an abrupt curvature greater than 60°, with an exponential increase of the gap in terms of TtF in relation to the increase of degrees of curvature. Considering the results and the above, the null hypothesis has been partially rejected.

However, this finding cannot be generalized since the instruments used in this research are characterized by a flat design that leads to a great reduction in terms of metal mass (nearly 50%). Moreover, in combination with the flat design, the S italic cross-sectional design guarantees a great reduction of the instrument mass and volume [22,24,25]. It is reasonable to suppose that increasing the metal mass of an instrument the gap between austenitic and martensitic instruments in terms of torsional resistance could be statistically detectable even in curvatures with fewer degrees, with a higher torsional resistance for austenitic instruments. For this reason, further research on this theme is needed.

Although the study assessed for the first time the relationship between the crystallographic phase of NiTi rotary instruments and the torsional resistance during different bending conditions, it has been done through a limited static investigation; thus, in order to clearly understand the real behavior of NiTi instruments with different crystallographic organization during instrumentation of root canal system, further research is needed, considering the topic in a dynamic view and taking into account the different cutting efficiency and stiffness conditions of the instruments. In fact, the current study focused on a specific condition by trying to isolate variables and determining an initial investigation on the relationship between the crystallographic phase and the torsional behavior of NiTi instruments in different bending conditions. However, as demonstrated by Silva et al., the clinical relevance of these mechanical tests is considered to be low because clinical usage can be affected by several other factors. As such, a multimethod approach should be recommended for further research with the aim to combine the results obtained from this study with the shaping ability of NiTi instruments with a different crystallographic phase in order to better interpret of their performance and, consequently, to carry out a more precise translation of preclinical findings to guide clinical use [26,27].

## 5. Conclusions

In conclusion, given the limitation of this study, it can be stated that the crystallographic phase of NiTi endodontic rotary instruments influences their maximum torque at fracture when they are subjected to severe bending moment. Moreover, it can be affirmed that when increasing the degrees of curvature and reducing the radius of curvature, the maximum torque at fracture of NiTi endodontic rotary instruments increases.

## Figures and Tables

**Table 1:** Summary of the results of TtF and FL with mean values and standard deviations according to the different bending conditions and crystallographic phases. The angle and the radius of curvature are specified in brackets. (Sg = Subgroup).

	F-One 20.04 Austenitic (Group A)					F-One 20.04 Martensitic (Group B)				
	Sg A1 (60°-5 mm)	Sg A2 (60°-3 mm)	Sg A3 (90°-5 mm)	Sg A4 (90°-3 mm)	Sg A5 (Straight)	Sg B1 (60°-5 mm)	Sg B2 (60°-3 mm)	Sg B3 (90°-5 mm)	Sg B4 (90°-3 mm)	Sg B5 (Straight)
TtF (Ncm)	0.54 ± 0.04	0.73 ± 0.05	0.96 ± 0.07	1.06 ± 0.07	0.42 ± 0.07	0.61 ± 0.09	0.71 ± 0.08	0.85 ± 0.04	0.93 ± 0.02	0.45 ± 0.02
FL (mm)	3.01 ± 0.04	3.03 ± 0.06	2.98 ± 0.05	3.04 ± 0.05	3.00 ± 0.06	2.96 ± 0.04	3.01 ± 0.04	3.02 ± 0.07	2.99 ± 0.06	3.06 ± 0.07

## Data Availability

Data sharing available upon contacting the corresponding author.
